# Matrotrophic viviparity constrains microbiome acquisition during gestation in a live‐bearing cockroach, *Diploptera punctata*

**DOI:** 10.1002/ece3.5580

**Published:** 2019-08-22

**Authors:** Emily C. Jennings, Matthew W. Korthauer, Trinity L. Hamilton, Joshua B. Benoit

**Affiliations:** ^1^ Department of Biological Sciences University of Cincinnati Cincinnati Ohio; ^2^ Plant and Microbial Biology and the BioTechnology Institute College of Biological Sciences University of Minnesota St. Paul Minnesota

**Keywords:** *Diploptera punctata*, insect, live birth, microbiome, reproduction, vertical transmission, viviparity

## Abstract

The vertical transmission of microbes from mother to offspring is critical to the survival, development, and health of animals. Invertebrate systems offer unique opportunities to conduct studies on microbiome‐development‐reproduction dynamics since reproductive modes ranging from oviparity to multiple types of viviparity are found in these animals. One such invertebrate is the live‐bearing cockroach, *Diploptera punctata*. Females carry embryos in their brood sac, which acts as the functional equivalent of the uterus and placenta. In our study, 16S rRNA sequencing was used to characterize maternal and embryonic microbiomes as well as the development of the whole‐body microbiome across nymphal development. We identified 50 phyla and 121 classes overall and found that mothers and their developing embryos had significantly different microbial communities. Of particular interest is the notable lack of diversity in the embryonic microbiome, which is comprised exclusively of Blattabacteria, indicating microbial transmission of only this symbiont during gestation. Our analysis of postnatal development reveals that significant amounts of non‐Blattabacteria species are not able to colonize newborn *D. punctata* until melanization, after which the microbial community rapidly and dynamically diversifies. While the role of these microbes during development has not been characterized, Blattabacteria must serve a critical role providing specific micronutrients lacking in milk secretions to the embryos during gestation. This research provides insight into the microbiome development, specifically with relation to viviparity, provisioning of milk‐like secretions, and mother–offspring interactions during pregnancy.

## Graphical abstract

Invertebrate systems offer unique opportunities to conduct studies on microbiome‐development‐reproduction dynamics since reproductive modes ranging from oviparity to multiple types of viviparity are found in these animals. One such invertebrate is the live‐bearing cockroach, and *Diploptera punctata* 16S rRNA sequencing was used to characterize the *D. punctata* maternal and embryonic microbiomes as well as the development of the whole‐body microbiome across nymphal development, identifying 50 phyla and 121 classes overall and found that symbionts are not transmitted to offspring during pregnancy, rather the microbiome is acquired during the first instar stage.

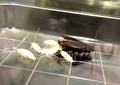

## 1 INTRODUCTION

Animals share their bodies with a diverse suite of microorganisms known as the microbiome (Engel & Moran, 2013; The NIH HMP Working Group, 2009). These microbes have important roles in a variety of processes benefiting their host, ranging from nutrient metabolism to immunity (Albenberg & Wu, 2014; Chung et al., 2012; Dimmitt et al., 2010; Douglas, 2017; Jašarević, Rodgers, & Bale, 2015; Michalkova, Benoit, Weiss, Attardo, & Aksoy, 2014; Pais, Lohs, Wu, Wang, & Aksoy, 2008; Snyder & Rio, 2015; Wang, Weiss, & Aksoy, 2013; Weiss, Wang, & Aksoy, 2011). For most animals, their microbial community is established over development through interactions with the environment, through diet, as well as interactions with other organisms (Abdul Rahman et al., 2015; Blaser & Dominguez‐Bello, 2016; Carrasco et al., 2014; da Costa & Poulsen, 2018; Estes et al., 2013; Funkhouser & Bordenstein, 2013; Gilbert, 2014; Korpela et al., 2018; Kostic et al., 2015; Morse et al., 2013; Mueller, Bakacs, Combellick, Grigoryan, & Dominguez‐Bello, 2015; Perez‐Muñoz, Arrieta, Ramer‐Tait, & Walter, 2017; Schwab, Riggs, Newton, & Moczek, 2016; Shukla, Vogel, Heckel, Vilcinskas, & Kaltenpoth, 2018; Torrazza & Neu, 2011; Wang & Rozen, 2017). Of interest is the role that parent–offspring interactions play in the microbial acquisition during early development, specifically from mother to her offspring (Adair & Douglas, 2017; Dimmitt et al., 2010; Duranti et al., 2017; Fox & Eichelberger, 2015; Funkhouser & Bordenstein, 2013; Gilbert, 2014; Jašarević, Rodgers, et al., 2015; Korpela et al., 2018; Kostic et al., 2015; Perez‐Muñoz et al., 2017; Schwab et al., 2016; Torrazza & Neu, 2011; Wade, 2014; Walker, Clemente, Peter, & Loos, 2017).

The animal's reproductive mode, in part, mediates the types of interactions mothers have with their offspring. Egg‐laying (oviparous) organisms have limited opportunity to pass microbes to offspring before they are born through hatching (Abdul Rahman et al., 2015; Bright & Bulgheresi, 2010; da Costa & Poulsen, 2018; Estes et al., 2013; Funkhouser & Bordenstein, 2013; Salem, Florez, Gerardo, & Kaltenpoth, 2015; Schwab et al., 2016; Shukla et al., 2018). This forces vertical symbiont transmission to occur through incorporation during oogenesis or by inoculating the external egg surface for consumption immediately upon juvenile emergence (Abdul Rahman et al., 2015; Estes et al., 2013; Funkhouser & Bordenstein, 2013; Schwab et al., 2016; Shukla et al., 2018). Viviparous (live‐bearing) animals can have extensive and complex interactions between mother and offspring during gestation and birth, the impacts of which can last for a few days to years (Cao‐Lei et al., 2017, 2014; Duranti et al., 2017; Funkhouser & Bordenstein, 2013; Jašarević, Rodgers, et al., 2015; Jiménez‐Chillarón et al., 2015; Ma et al., 2014; Ogawa & Miura, 2014; Poulin & Thomas, 2008; Stein & Lumey, 2000; Torrazza & Neu, 2011; Weiss et al., 2011). These prolonged interactions provide means for multiple routes of vertical transmission of microbes from mother to her progeny (Funkhouser & Bordenstein, 2013; Ma et al., 2014; Mueller et al., 2015). In humans, while placental transmission of microbes is debated (Aagaard et al., 2014; Blaser & Dominguez‐Bello, 2016; Fardini, Chung, Dumm, Joshi, & Han, 2010; Perez‐Muñoz et al., 2017; Walker et al., 2017), mother to newborn transfer can occur during passage through the birth canal, breast feeding, and throughout early postnatal development (Ballard & Morrow, 2013; Dahlen, Downe, Kennedy, & Foureur, 2014; Duranti et al., 2017; Funkhouser & Bordenstein, 2013; Jašarević, Howerton, Howard, & Bale, 2015; Jašarević, Rodgers, et al., 2015; Korpela et al., 2018; Ma et al., 2014; Mueller et al., 2015). Other live‐bearing animals and their symbionts have evolved to utilize the extended gestation as a time to inoculate progeny with bacteria (Denlinger & Ma, 1975; Funkhouser & Bordenstein, 2013; Ma et al., 2014; Morse et al., 2013; Mueller et al., 2015; Wang et al., 2013). This is exemplified in tsetse flies and other members of Hippoboscoidea, where mothers utilize nutritive secretions as a mechanism to transfer required symbiotic bacteria to their intrauterine developing larvae (Douglas, 2017; Morse et al., 2013; Snyder & Rio, 2015; Wang et al., 2013; Weiss et al., 2011). For tsetse flies, symbiotic bacteria, specifically *Wigglesworthia*, provide key B vitamins that are low in their food source (blood) or within milk transferred to the developing intrauterine larva and are critical to immune function (Akman et al., 2002; Attardo et al., 2019; Benoit et al., 2017; Rio et al., 2012). Here, we examine shifts in the microbiome of the live‐bearing cockroach, *Diploptera punctata*, during pregnancy and development.

**Figure 1:**
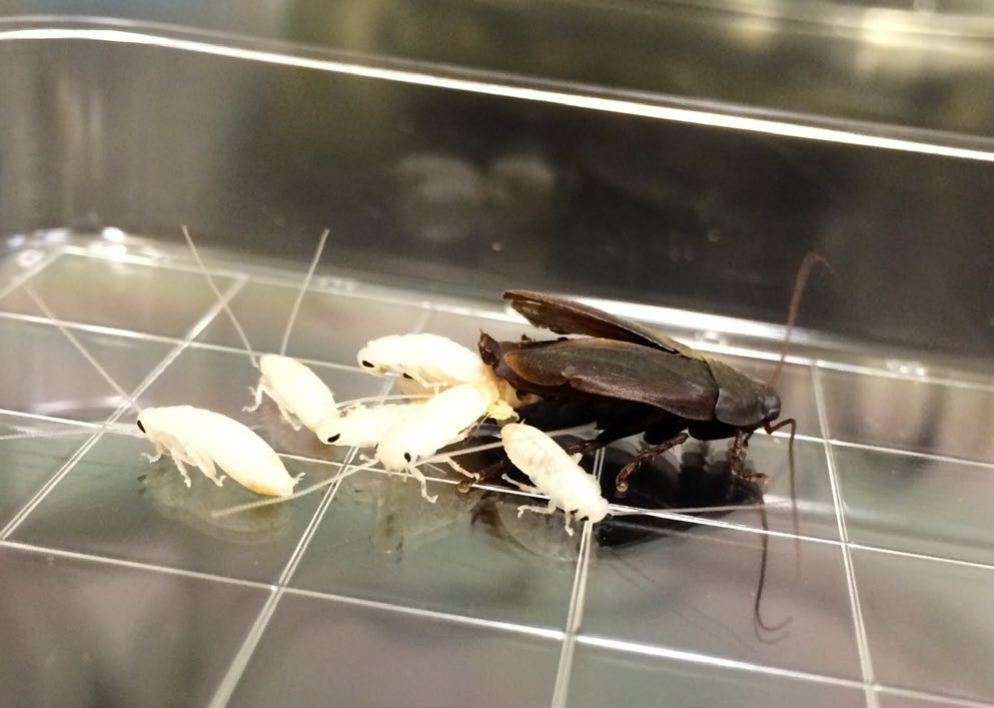
*Diploptera punctata* reproduce by matrotrophic viviparity, this female *D. punctata* is giving birth, surrounded by her newly born nymphs

*Diploptera punctata* reproduces by matrotrophic viviparity (Figure 1), in which embryos develop inside the brood sac, a unique organ which functions as both a uterus and pseudo‐placenta, and are provided with nutrients by a secretion of milk‐like components (Hagan, 1939, 1941; Roth & Willis, 1955, 1957; Stay & Coop, 1973). This secretion appears in embryo gut contents at 20% of the 60–70‐day pregnancy, when the dorsal edge of the body wall is closed (Ingram, Stay, & Cain, 1977; Roth & Willis, 1955; Stay & Coop, 1973, 1974). *Diploptera* milk is a combination of proteins and free amino acids, carbohydrates, and lipids in a water base (Ingram et al., 1977; Stay & Coop, 1974; Williford, Stay, & Bhattacharya, 2004; Youngsteadt, Fan, Stay, & Schal, 2005). The proteins present include a unique family of lipocalin‐like milk proteins (Ingram et al., 1977; Stay & Coop, 1974; Williford et al., 2004). While this milky secretion provides vital nutrients to developing embryo, it is deficient in two essential amino acids, methionine and tryptophan (Ingram et al., 1977; Williford et al., 2004). It has been proposed that bacterial endosymbionts provide these two nutrients (Williford et al., 2004); however, in oviparous cockroaches the only bacterium transmitted from mother to embryo belongs to the Flavobacteria family Blattabacteriaceae (Bandi et al., 1994, 1995; Giorgi & Nordin, 1994). Most, but not all, strains of Blattabacteria have an incomplete biosynthetic pathway for methionine (Huang, Sabree, & Moran, 2012; Kambhampati, Alleman, & Park, 2013; López‐Sánchez et al., 2008, 2009; Patiño‐Navarrete, Moya, Latorre, & Peretó, 2013; Sabree, Kambhampati, & Moran, 2009; Tokuda et al., 2013). This leads us to the question, do *D. punctata* embryos inherit only Blattabacteria, capable of methionine biosynthesis, from their mothers, or does the extended association between mother and offspring allow colonization of the embryonic microbiome by additional bacteria?

To address this question, this study determined the microbiome of *D. punctata* throughout development, characterizing the microbial communities inhabiting female *D. punctata* and their offspring across development using 16S rRNA gene sequencing. The information generated by this study will provide the first step in developing *D. punctata* as a model system to elucidate how intrauterine development and the prenatal microbiome affect later acquisition of microbial endosymbionts. Developing a new model system understanding microbial shifts during invertebrate matrotrophic viviparity will widen the evolutionary lens through which we view reproduction and the microbiome in viviparous animals.

## 2 METHODS

### 2.1 Animals

Colonies reared at the University of Cincinnati (UC) Department of Biological Sciences (Cincinnati, OH) were housed in a climate‐controlled facility. Ambient temperature was held between 24–28°C, and relative humidity (RH) was held between 70%–80%. A 12:12‐hr light–dark photoperiod was maintained for the duration of the experiment. Animals were provided water and fed Old Roy Complete Nutrition brand dog food (Mars, Inc.) ad libitum. A second group of *D. punctata* were obtained from The Ohio State University (OSU) Biological Sciences Greenhouse (Columbus, OH) insect collection where they were reared in similar conditions with the exception of being fed a diet of Tetramin fish food (Spectrum Brands Pet). This second group was collected randomly from the OSU colony and brought to the UC laboratory, where they were housed separately from the UC colony under identical conditions and provided the same food and water sources as the UC colony for 1 week, when sacrificed for sample collection.

### 2.2 Sample collection

Visibly pregnant females were selected from the colony for use in mother–embryo comparisons. Females were surface sterilized by rinsing for 1 min in each of the following solutions: 70% ethanol and 2% sodium hypochlorite. This was followed by four rinses in sterile phosphate‐buffered saline (PBS; 81 mM Na_2_HPO_4_, 19 mM NaH_2_PO_4_, 150 mM NaCl, pH 7.4). Embryo broods were then dissected from the brood sac in sterile PBS by making two small incisions at the opening of the brood sac, one on each side, and removed using ethanol sterilized forceps. To determine the developmental stage of the embryos, a single embryo from the center of each brood was measured on a bleach sterilized ruler and designated as prelactation, early lactation, or late lactation based on its length (Table 1; Stay & Coop, 1973). Entire broods of embryos and individual mothers were then placed into separate 1.5‐ml centrifuge tubes with silica beads and stored at −80°C until processing. While mother–embryo pairs were collected for all three trimesters, only late lactation pairs were utilized in this study. Nine mother–embryo pairs were collected from the UC colony for analysis and 12 from the OSU colony.

**Table 1:** Pregnancy stage determination

Reproductive stage	Embryo length	Estimated embryo age	
Not Pregnant (NPF)	Not present	n/a	
PreLactation (PreL)	<1.6 mm	0–11 days	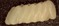
Early Lactation (EarL)	1.6–2.5 mm	12–27 days	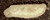
Late Lactation (LateL)	>2.5 mm	28–55 + days	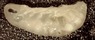

Note

This table describes the measurements utilized to determine pregnancy stage based on a previous study by Stay and Coop (1973).

To characterize the postnatal development of the microbiome, visibly pregnant females were again selected from the colony and housed in individual containers with food and water ad libitum and monitored for active birthing. Nymphs were collected as neonates (identified by lack of cuticular melanization) or first instars (identified by melanization within 12 hr of birth). Second‐, third‐, and fourth‐instar nymphs were sampled and identified by size and the presence of molts in the living quarters. Postnatal samples were collected only from the UC colonies. Upon collection, nymphs were surface sterilized using the methods described above and then stored in 1.5‐ml centrifuge tubes with silica beads at −80°C until processing. Five neonates, seven first instars, nine second instars, nine third instars, and six fourth instars were utilized in this analysis.

### 2.3 Genomic DNA preparation

Samples were homogenized in 1 µl of sterile 1× PBS, and DNA was extracted using a QIAGEN DNeasy Blood and Tissue Kit (Qiagen). The homogenate (200 µl) was incubated with proteinase K (Qiagen) over night before continuing the provided protocol. DNA concentration and quality were measured using a NanoDrop 2000. All samples were diluted to 20 ng/µl for sequencing.

### 2.4 16S rRNA sequencing and bioinformatic analyses

The V4 hypervariable region of the bacterial 16S rRNA gene was PCR amplified using the 515f (GTGYCAGCMGCCGCGGTAA) and 806r (GGACTACNVGGGTWTCTAAT) universal primers (Apprill, McNally, Parsons, & Weber, 2015; Caporaso et al., 2011). Amplicon sequencing using the MiSeq Illumina 2 × 300 bp chemistry was conducted at the Miami University Center for Bioinformatics & Functional Genomics (Oxford, OH, USA) as well as the University of Minnesota Genomics Center (Minneapolis, MN, USA).

Using the Ohio Supercomputer Center resources (Ohio Supercomputer Center, 1987), sequence reads were processed in mothur (v.1.39.5; Schloss et al., 2009) based on the published MiSeq SOP (Kozich, Westcott, Baxter, Highlander, & Schloss, 2013). Briefly, the make.contigs command was used to extract quality data from the reads and only reads possessing a quality score greater than 25 were joined to make the contigs for further analysis. Screen.seqs was utilized to remove contigs containing ambiguous bases, contigs longer than 275 bp, and those containing homopolymers longer than 8 bp. Unique.seqs and count.seqs were utilized to remove duplicate sequences and generate count tables. Taxonomic assignment of sequences was conducted using align.seqs to compare the contigs to the SILVA database (v.123; Quast et al., 2013) containing only the V4 region aligning with the primers used. Filter.seqs was used to remove sequences that have large gaps in the alignments. Chimeric sequences were removed using the UCHIME (Edgar, Haas, Clemente, Quince, & Knight, 2011) algorithm using the chimers.uchime and remove.seqs commands. Non‐16S rRNA gene sequences were removed using the classify.seqs and remove.lineage commands. Sequences were clustered using the cluster.split command at the taxonomic level 4, representing order. All further analyses were conducted using operational taxonomic unit (OTU) assignments generated in the above steps. Rarefaction curves were generated using the rarefaction.single and the number of observed OTUs (sobs), demonstrating adequate sequencing depth (Table S1). Alpha diversity was assessed using the inverse Simpson, and Shannon diversity metrics. NMDS and PCOA analyses were conducted using mothur. Community composition was manually assessed for visualization at taxonomic level 5, representing bacterial families. Linear discriminant analysis effect size (LEfSe) as implemented in mothur was utilized to identify stage‐specific OTUs across development (Segata et al., 2011); a p‐value cutoff of 0.01 was utilized. In addition to mothur, we performed a second analysis of our data for validation purposes utilizing QIIME (v. 1.9.1; Caporaso et al., 2010) as implemented by the Nephele pipeline (v. 2.2.2; Weber et al., 2018) using the default settings, referencing the SILVA database (v.128 SSU REF 99; Quast et al., 2013). When relative abundances calculated at the class level by both methods were compared, they were found to be significantly correlated (Figure S1); consequently, results from mothur were reported. Additional results from the QIIME analysis can be found in Data S1 and Data S2.

Data processing was conducted in Microsoft Excel (v.16.22) and R (v.3.3.3; R Core Team, 2017) using RStudio (v1.1.423; RStudio Team, 2015). Additional statistics and graphical representations of data were also performed in R using RStudio. Packages utilized include dplyr (Wickham, Francois, Henry, & Müller, 2019), dunn.test (Dinno, 2017), ggplot2 (Wickham, 2016), reshape2 (Wickham, 2007), RColorBrewer (Neuwirth, 2014), Rmisc (Hope, 2013), and wesanderson (Ram & Wickham, 2018).

## 3 RESULTS

### 3.1 Maternal and embryonic microbiomes

**Figure 2:**
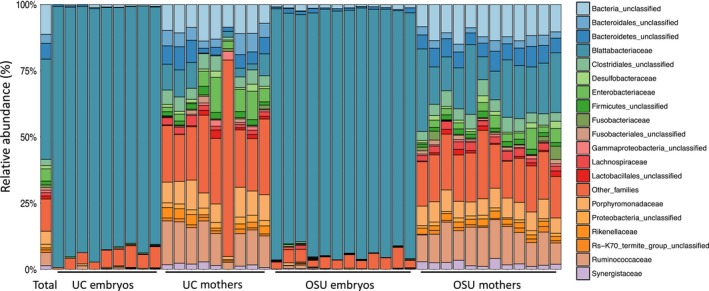
Embryo microbiomes from both the University of Cincinnati (UC) and Ohio State University (OSU) colonies are dominated by the family *Blattabacteraceae* while mothers are more diverse. Relative abundances of the 19 most abundant bacterial families in *Diploptera punctata* mothers and embryos. The remaining families are cumulatively represented as “other”. The *y*‐axis represents the percent of total OTUs present in each sample for each family. Each bar represents an individual mother or brood of embryos

Amplicons from the 16S rRNA generated 2,180,632 paired‐end reads from both OSU and UC colony mothers and embryos of *D. punctata*, assembled into 2,170,187 contigs when joined. Of those, 1,759,259 total sequences passed quality control and were classified as archaea (8,320 reads; 0.473%), bacteria (1,750,772 reads; 99.518%), or unknown (167 reads; 0.009%; Table S2). Removal of unwanted classifications (archeae, chloroplast, eukaryote, mitochondria, and unknown) yielded 1,749,921 merged reads, ultimately generating 38,969 bacterial operational taxonomic units (OTUs) corresponding to 44 phyla, 108 classes, 204 orders, 386 families, and 710 genera. Overall, Bacteroidetes was the most prominent phylum (21,099 OTUs; 54.143%), followed by Firmicutes (5,513 OTUs; 14.147%), Proteobacteria (4,783 OTUs; 12.274%), and unclassified bacteria (4,286 OTUs; 10.998%; Figure 2). At the family level, Blattabacteriaceae, a family of Flavobacteria, was the most represented overall in both OTUs (14,426 OTUs; 37.019%) and reads (1,038,785 reads; 59.047% of all reads including nonbacterial) with unclassified bacteria being the next most abundant family (4,286 OTUs; 10.998%) followed by unclassified Bacteroidetes (2,260; 5.799%) and Ruminococcaceae (1,890; 4.850%; Figure 2, Table S3).

**Figure 3:**
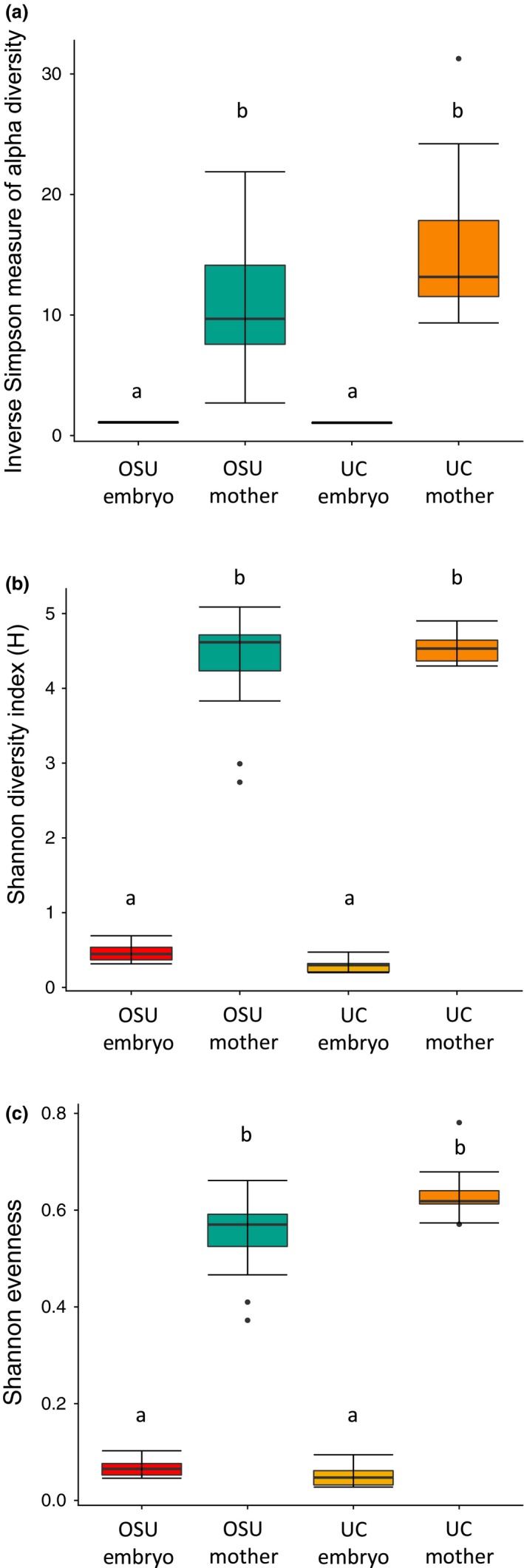
Microbiomes of *Diploptera punctata* mothers and embryos differ significantly in measures of diversity and evenness; embryo samples are significantly less diverse and even than mothers regardless of colony origin. Measure of diversity and evenness calculated using mothur for mothers and embryos of both UC and OSU colonies. (a) Inverse Simpson measure of alpha diversity (b) Shannon's diversity index (c) Shannon's evenness index. Median value is represented as the center line of each box while the lower and upper limits of the box represent the 25th and 75th quantiles, respectively. Error bars extend to the last data point within the hinge value ± 1.5* the interquartile range. Significance determined by Kruskal–Wallis and Dunn's test, alpha = 0.025

**Figure 4:**
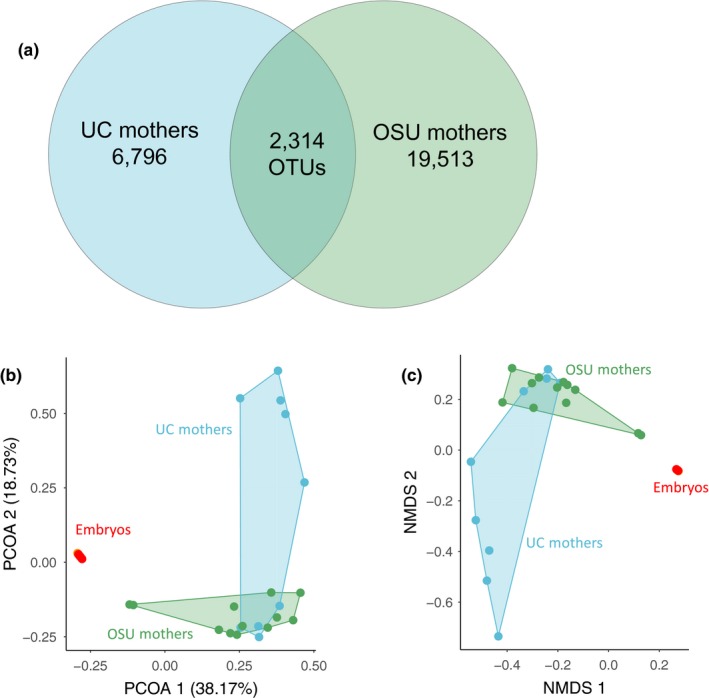
*Diploptera punctata* mothers share a 2,314 OTU core microbiome but form unique clusters based on colony origin in ordination analyses of communities, while embryos form a single cluster regardless of origin. Community comparisons between *D. punctata* mothers and embryos of both colonies (a) Number of OTUs recovered for mothers of the UC and OSU colonies. A 2,314 OTU core component of the maternal microbiome was identified using mothur. (b) Principle coordinate analysis [PCOA] of mothers and embryos from both colonies. (c) Nonmetric multidimensional scaling [NMDS] of mothers and embryos from both colonies. In (b) and (c), embryos cluster so closely that the samples are indistinguishable

In mothers, OTUs were distributed among the same top four phyla (Bacteroidetes, 35.354%; Firmicutes, 27.714%; Proteobacteria, 14.138%; unclassified bacteria, 11.609%), with a similar distribution among mothers of both the OSU and UC colonies. At the family level, OTUs derived from *D. punctata* mothers were most represented in Blattabacteriaceae (6,734; 13.842%), Ruminococcaceae (5,781; 11.883%), and unclassified bacteria (5,648; 11.609%; Figure 2). Mothers from the OSU and UC colonies had similar distributions of OTUs among families. Additionally, there was no significant difference between the two colonies in community diversity or evenness (Figure 3). We identified a core community of 2,314 OTUs shared between mothers of both colonies, composed of 25 phyla with Firmicutes and Bacteroidetes representing more than 60% of OTUs (Figure 4, Table S4). No individual family represented more than 16% of the core OTUs, with Ruminococcaceae (16%) being the most abundant of the top eight families (52%; Table S4).

Approximately 89% of OTUs and 99% of sequencing reads from embryos of both colonies belonged to the family Blattabacteriaceae, while all other families individually represented 1% or less of OTUs and 0.08% of embryo‐derived sequencing reads (Figure 2, Figure S2). Additionally, it should be noted that these low abundance taxa show no consistency in representation across embryo samples with varying numbers of reads and OTUs (Tables S2 and S3). These findings were corroborated by secondary analyses completed using the Nephele implementation of QIIME, despite inherent differences in computational methods (Data S1). Embryos of both UC and OSU colonies did not differ significantly in diversity, evenness, and species richness (Figure 3). However, microbial communities of embryos were less diverse and less so than mothers across both colonies (Figure 3). Analysis of molecular variance in mothur revealed that despite our four sampling groups consisting of mothers and embryos from two distinct colonies, there exist three distinct subcommunities corresponding to UC mothers, OSU mothers, and all embryos (Figure 4, Table S5).

While the transmission of the cockroach‐specific endosymbiont Blattabacteria is known to occur during oogenesis (Sacchi et al., 1996), surface sterilization of oothecae, and hatching into a sterile environment results in a microbiome exclusively composed of Blattabacteria, indicating any other bacteria must be acquired from food or feces (Pietri, Tiffany, & Liang, 2018). Such is the case in the intergenerational transfer of microbiota via proctodeal trophallaxis in *Cryptocercus punctulatus* and *Mastotermes darwiniensis* (McMahan, 1969). Because *D. punctata* harbor their developing embryos for their gestational period, it is possible other bacteria may be transmitted via the brood sac. The low diversity and overall OTUs present in embryonic samples, however, suggest that if other bacteria are transmitted during gestation, the number is very low and is not likely of significance to *D. punctata* embryos. This indicates that Blattabacteria are the main endosymbiont during intrauterine development in *D. punctata* and that any additional constituents of the microbiome colonize after birth.

### 3.2 Postnatal microbiome development

We next sought to determine the progression of the microbiome over postnatal development. Because we found no significant differences between the OSU and UC colonies of *D. punctata*, the samples were recategorized for subsequent analyses and denoted simply as mothers and embryos. To determine the succession of the microbial communities inhabiting *D. punctata* from embryo to adulthood, we surveyed the microbiome of neonate nymphs and each of the following nymphal instars (one through four).

**Figure 5:**
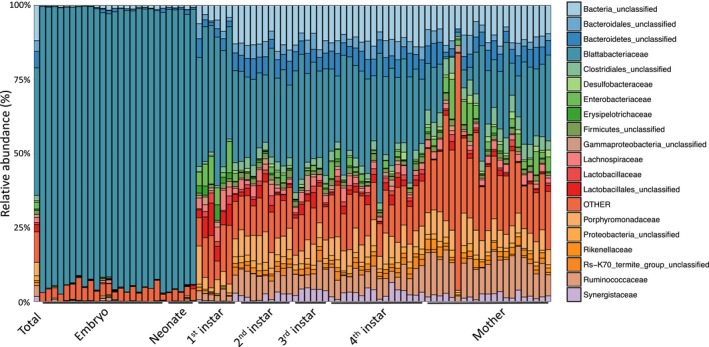
Embryos and newborn *Diploptera punctata* microbiomes are dominated by Blattabacteriaceae while first instars and beyond have microbial communities contain a greater number of highly represented bacterial families. Relative abundances of the 19 most abundant bacterial families in *D. punctata* embryos, nymphs, and adult females. The *y*‐axis represents the percent of total OTUs present in each sample for each family. Each bar represents an individual sequencing replicate; nymphs and mothers were individual animals while embryos were whole broods

A total of 6,453,793 paired reads from mothers, embryos, and juvenile instars were used to generate 6,443,348 contigs in mothur. Of these, 4,752,552 passed quality control and were able to be taxonomically classified as either archeae (14,141; 0.298%), bacteria (4,737,007; 99.673%), or unknown (1,402; 0.029%; Table S6). Removing unwanted reads as before, 4,734,605 remained and were utilized to generate 209,554 bacterial operational taxonomic units (OTUs) including 50 phyla, 122 classes, 252 orders, 485 families, and 1,008 genera (Table S7). As expected, Bacteroidetes was again the most abundant phylum (122,945 OTUs; 58.670%) when all samples were combined, followed by Firmicutes (29,705 OTUs; 14.175%), unclassified bacteria (24,777 OTUs; 11.824%), and Proteobacteria (20,068 OTUs; 9.577%). Flavobacteria and unclassified bacterial classes comprised 54.979% of class‐level OTUs, a trend that holds true at the order level as well (Table S7). At the family level, Blattabacteriaceae (42.877%) was again the most prominent taxon followed by unclassified bacteria (11.824%), unclassified Bacteroidetes (5.515%), and Porphyromonadaceae (4.506%; Figure 5, Table S7).

Blattabacteriaceae (93.387%), again, were the primary constituent and defining feature of the embryonic microbial community, while other families each represented 0.716% or less of the OTUs present (Figure 5, Tables S7 and S8). The dominance of the microbial community by Blattabacteriaceae persisted after birth during the neonate stage (93.741%) with each other family representing less than 1% of the community (Figure 5, Table S7). Of the eight OTUs identified as enriched in neonates, seven corresponded to Blattabacteriaceae and only one was representative of Streptococcaceae (Table S8). Postmelanization first instars, however, have a more diverse microbial community, and we identified 58 enriched OTUs corresponding to 31 families (Table S8). While Blattabacteriaceae is still the most abundant family (40.442%), a significant portion of the community (a combined 23.494% of OTUs) is made up by unclassified bacteria (6.281%), Enterobacteriaceae (6.125%), unclassified Lactobacillales (5.737%), and Porphyromonadaceae (5.351%), while all other families individually represented less than 4% of the first‐instar microbial community (Figure 5, Table S7). Of the 31 enriched families we identified in first‐instar samples, the family Lachnospiraceae is the most represented (10 OTUs) while Blattabacteriaceae is among the lowest represented (1 OUT; Table S8). Second instars had more families represented in high levels. Blattabacteriaceae represented only 28.303% of the community, while unclassified bacteria (12.978%), Porphyromonadaceae (7.728%), Ruminococcaceae (7.705%), and unclassified Bacteroidetes (5.854%) increased in representation and together make up 34.265% of the OTUs. This expansion of the microbiome is reflected in an increased number of enriched OTUs and associated families, and 137 enriched OTUs belonging to 44 families were identified. Ruminococcaceae, with 39 representative OTUs, is a key taxon defining the second‐instar microbial community with no enriched OTUs corresponding to Blattabacteriaceae (Table S8). This redistribution of abundance from Blattabacteriaceae is maintained after the second‐instar stage, with abundances in third and fourth instars remaining around 30% and no representation in the enriched OTUs (Figure 5, Tables S7 and S8). In third instars, Ruminococcaceae (10 OTUs) is also the most represented family in the 67 enriched OTUs, followed by Synergistaceae (6 OTUs; Table S8). In the 105 fourth‐instar‐specific OTUs, Ruminococcaceae and Porphyromonadaceae were the two most represented families, each with 11 OTUs followed by Synergistaceae with 8 OTUs (Table S8). Adult females had even lower abundances of Blattabacteriaceae, although it was still the most abundant family (18.826%). All families represented less than 20% of the OTUs present, with Ruminococcaceae and unclassified bacteria being the only two with abundances greater than 10% (Figure 5, Table S7). The 205 mother‐enriched OTUs represented 61 families, predominantly Ruminococcaceae (48 OTUs) followed by unidentified Clostridiales (21 OTUs) and Porphyromonadaceae (14 OTUs; Table S8). Again, no enriched OTUs corresponded to Blattabacteriaceae.

**Figure 6:**
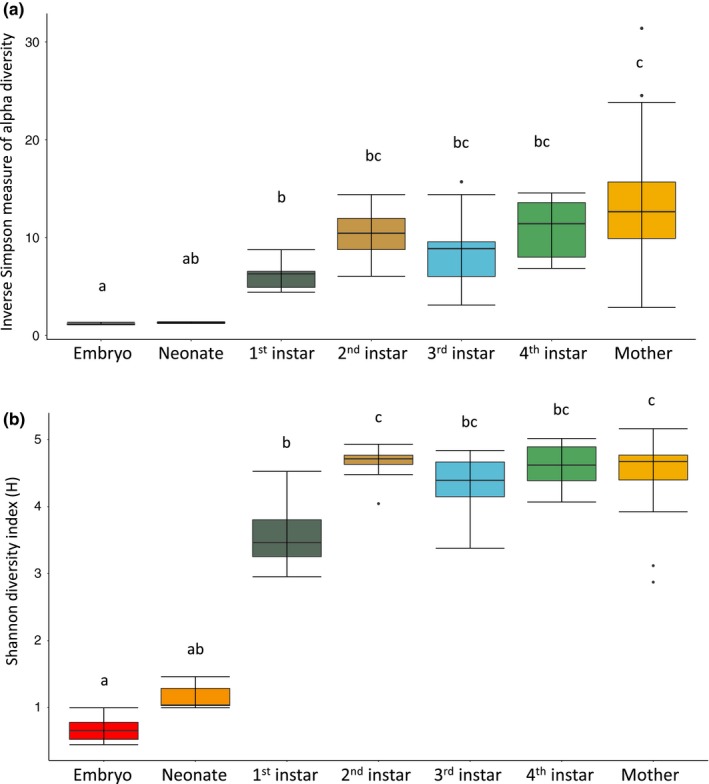
Diversity of the microbial community in *Diploptera punctata* increases significantly from birth to adulthood. Measures of diversity and evenness generated by mothur for embryos, all nymphs, and adult females of *D. punctata* (a) Inverse Simpson (b) Shannon's diversity index. Median value is represented as the center line of each box while the lower and upper limits of the box represent the 25th and 75th quantiles, respectively. Error bars extend to the last data point within the hinge value ± 1.5* the interquartile range. Significance determined by Kruskal–Wallis and Dunn's test, alpha = 0.025

Multiple measures of diversity varied across the life stages of *D. punctata*. While embryos and neonates did not differ in either the Shannon index or Inverse Simpson, all other instars and mothers were significantly different from embryos in both measures (Figure 6). Neonates also did not differ from first instars but showed significant differences in both diversity metrics compared to second, third, and fourth instars as well as adult females. Second, third, and fourth instars, however, did not differ from each other or mothers in any diversity measure (Figure 6). While AMOVA and HOMOVA analyses revealed slightly different relationships between the samples (Table S9), the analyses consistently showed that embryos and neonates differed from the other juvenile stages and adult females. These results further support our hypothesis that *D. punctata* acquire microbial endosymbionts (outside of Blattabacteria), not through direct maternal transfer during gestation but in the days and weeks after birth, primarily during and after initial melanization during the first nymphal instar.

## 4 DISCUSSION

We identified 50 phyla, 122 classes, 252 orders, 485 families, and 1,008 genera as part of the overall *D. punctata* microbial community. Our analyses revealed that Bacteroidetes, Firmicutes, and Proteobacteria were the dominant phyla in addition to unclassified bacteria. Previous studies have characterized microbial communities of cockroaches, primarily the gut microbiome. Consistent with our findings, Bacteroidetes, Firmicutes, Proteobacteria, and unclassified bacteria are repeatedly found to be prominent members of adult cockroach endosymbiont communities (Bauer et al., 2015; Bertino‐Grimaldi et al., 2013; Carrasco et al., 2014; Gontang et al., 2017; Kakumanu, Maritz, Carlton, & Schal, 2018; Pérez‐Cobas et al., 2015; Schauer, Thompson, & Brune, 2014; Tinker & Ottesen, 2016). Similar to our adult female samples, other studies have shown Porphyromonadaceae, Rikenellaceae, and Bacteroidaceae to be the most abundant families of Bacteroidetes; while Lachnospiraceae, Ruminococcaceae, Clostridiaceae, and Lactobacillaceae are commonly represented Firmicutes (Bauer et al., 2015; Bertino‐Grimaldi et al., 2013; Carrasco et al., 2014; Gontang et al., 2017; Kakumanu et al., 2018; Pérez‐Cobas et al., 2015; Sabree & Moran, 2014; Schauer et al., 2014; Tinker & Ottesen, 2016). Proteobacteria present in cockroach microbiomes often belong to the families Desulfobacteraceae, Enterobacteriaceae, and Desulfovibrionaceae (Bauer et al., 2015; Bertino‐Grimaldi et al., 2013; Carrasco et al., 2014; Gontang et al., 2017; Kakumanu et al., 2018; Pérez‐Cobas et al., 2015; Sabree & Moran, 2014; Schauer et al., 2014; Tinker & Ottesen, 2016). Most previous cockroach microbiome studies found extremely low representation of Blattabacteria or do not report on its abundance due to the specific sampling of gut tissue; Blattabacteria reside in the fat body and ovaries and thus will be lacking in studies focus on the gut microbiome (Bauer et al., 2015; Bertino‐Grimaldi et al., 2013; Carrasco et al., 2014; Gontang et al., 2017; Kakumanu et al., 2018; Pérez‐Cobas et al., 2015; Sabree & Moran, 2014; Schauer et al., 2014; Tinker & Ottesen, 2016). The few studies that performed microbiome analyses on whole bodies or carcasses without guts, however, report Blattabacteriaceae abundances ranging from 8% to 90% depending on the habitat sampled, although carcasses without guts were generally found to contain predominantly Blattabacteria (Carrasco et al., 2014; Kakumanu et al., 2018).

Investigations of developmental acquisition of the cockroach microbiome are rare; however, one study characterized the succession of the microbiota in the oviparous German cockroach, *Blattella germanica* (Carrasco et al., 2014). Contents of surface‐sterilized oothecae contain exclusively Blattabacteria and whole bodies of first‐instar nymphs that hatched from unsterilized oothecae contain predominantly Blattabacteria, but have begun to acquire other gut symbionts (Carrasco et al., 2014). Despite the difference in reproductive mode, we found similar results in the intrauterine developing embryos and neonatal *D. punctata*.

One previous study has attempted to characterize the microbiome of *D. punctata* mothers and embryos, concluding that there are significant amounts of non‐Blattabacteria microbes in embryos (Ayayee, Keeney, Sabree, & Muñoz‐Garcia, 2017). In direct contrast, our embryo samples from two independent colonies, including the colony used in the previous study, produced sequencing reads that were 99.5% assigned to Blattabacteriaceae. Two taxa identified to be significantly enriched in the embryonic microbiome by this previous study were Halomonadaceae and Shewanellaceae (Ayayee et al., 2017), neither of which were present in our maternal, embryo, or postnatal development samples. While our analysis using mothur did identify non‐Blattabacteria sequences in embryonic samples, the extremely low abundances (<0.5% of total raw reads combined) suggest they are sequencing artifacts or misidentified and are not likely critical for embryos during gestation. This is further supported by our secondary analysis using the Nephele implementation of QIIME (Table S8, Data S1 and Data S2), which identified no taxa representing more than 0.2% of the embryonic community other than Blattabacteria. The fact that there is no consistency among low abundant taxa among embryo sample supports that bacteria, other than Blattabacteria, are not likely critical for the intrauterine stages. Because of our robust sampling method, including two separately housed colonies of *D. punctata* from separate institutional origins and use of two independent pipelines for analysis, we conclude that no bacterial transmission occurs after oogenesis during intrauterine development in *D. punctata*. Thus, Blattabacteria is the only bacterial component of the microbiome during intrauterine development. This is further supported by the lack of additional bacterial components in first‐instar nymphs collected immediately after birth (=neonate). While we cannot eliminate rearing differences, our study indicates that other bacteria, beyond Blattabacteria, are not required for *D. punctata* development.

After determining that there was no significant gestational transmission of endosymbionts, we sought to characterize the microbial community across nymphal development. *D. punctata* juveniles have a minimum of three nymphal instars with females molting an additional time to a fourth‐instar stage. Newborn, unmelanized first‐instar nymphs did not differ in bacterial community from intrauterine developing embryos suggesting that significant bacterial transmission does not occur during the birthing process, unlike humans. However, by the time first instars fully develop a hardened cuticle they have developed a more diverse microbial community where Blattabacteria represents only 35% of the OTUs. This substantial increase is likely the results of food and water consumption that occurs following melanization. Across the remaining instars, the community continues to become more diverse; however, the changes become much less dramatic after the second‐instar stage. These findings are again consistent with a previous study investigating the juvenile microbiome of *B. germanica* as well as in other egg‐laying organisms such as burying beetle *Nicrophorus vespilloides* (Carrasco et al., 2014; Wang & Rozen, 2017). Consequently, we conclude that the microbial community is largely acquired during the first‐ and second‐instar stages, likely from their environment where they cohabitate with both adult and other juvenile cockroaches, after they have started to feed and drink. There are continuously changes to the microbiome throughout the life of the animal, but these are minor compared to the initial acquisition in early developmental stages.

This initial acquisition period of the microbiome is extremely important to animal development (Albenberg & Wu, 2014; Ballou et al., 2016; Breznak & Kane, 1990; Brownlie & Johnson, 2009; Chung et al., 2012; Colston, 2017; Coon, Brown, & Strand, 2016; Coon, Vogel, Brown, & Strand, 2014; Diaz Heijtz, 2016; Dimmitt et al., 2010; Hamdi et al., 2011; Kostic et al., 2015; Lee & Brey, 2013; Ma et al., 2014; Malmuthuge, Griebel, & Guan, 2015; McFall‐Ngai, 2014; Michalkova et al., 2014; Pais et al., 2008; Pietri et al., 2018; Schwab et al., 2016; Snyder & Rio, 2015; Thompson, Rivera, Closek, & Medina, 2015; Torrazza & Neu, 2011; Wade, 2014; Yang et al., 2016). Studies in insect systems have demonstrated this by ablating the microbiome of juvenile animals and observing the phenotypes. Consistently, these experiments find that animals unable to acquire microbes from their environment or mothers face severe disadvantages, often failing to progress from one instar to the next, unable to molt to adulthood or undergo pupation, or dying. One example of this is the inability of axenic mosquito larvae to reach adulthood (Coon et al., 2016, 2014). In the dung beetle *Onthophagus gazella*, removal of a maternally provided fecal secretion, known as the pedestal, significantly reduces bacterial load in larvae hatched from surface‐sterilized eggs (Schwab et al., 2016). While preventing microbiome acquisition in *O. gazelle* larvae does not result in mortality as in mosquitoes, it is associated with reduced larval mass, increased time to adulthood, smaller adult body size, and impaired dehydration tolerance (Schwab et al., 2016). In tsetse flies, *Wigglesworthia glossinidia* transmission via milk gland secretions is not only essential for B vitamin provisioning, but also immune function by influencing the expression of a specific odorant binding‐protein (obp) in the larvae (Benoit et al., 2017; Weiss et al., 2011). Targeted elimination of this symbiont or the associated obp decreased the population of phagocytic hemocytes and reduced melanization ability (Benoit et al., 2017; Weiss et al., 2011). Symbiont community composition has also been implicated in insecticide resistance in the German cockroach (Pietri et al., 2018). Elimination of all bacteria from the cockroach except for Blattabacteria throughout development suggests that insecticide resistance are due to changes in non‐Blattabacteria bacteria which are acquired after hatching (Pietri et al., 2018). These studies underscore the importance of developing a diverse and robust microbial community during early nymphal development, which we have found primarily occurs during the first instar of *D. punctata*.

The embryonic microbiome comprised exclusively of Blattabacteria is of interest relative to the intrauterine development of *D. punctata* embryos, as the milk‐like secretion provided by mothers as the sole form of nutrition during development is largely devoid of two essential amino acids, methionine and tryptophan (Stay & Coop, 1974; Williford et al., 2004). Consequently, it has been suggested that these amino acids are acquired from bacterial endosymbionts (Williford et al., 2004). Bacterial symbionts commonly serve to supplement nutrients that may be lacking in the diet (Bermingham & Wilkinson, 2009; Douglas, 2017; Engel & Moran, 2013; Funkhouser & Bordenstein, 2013; Michalik, Szklarzewicz, Jankowska, & Wieczorek, 2014; Michalkova et al., 2014). Viviparous insects, such as tsetse flies, take advantage of endosymbionts to fill such nutritional gaps during development, mostly through the provisioning of B vitamins (Douglas, 2017; Snyder, Mclain, & Rio, 2012; Snyder & Rio, 2015; Wang et al., 2013). However, while *Wolbachia* is transmitted through the germ line before nutrient provisioning (Wang et al., 2013), other symbionts in these flies, such as *Wigglesworthia* and *Sodalis*, have been shown to be transmitted from mother to offspring during their extended gestation period (Denlinger & Ma, 1975; Douglas, 2017; Snyder et al., 2012; Snyder & Rio, 2015; Wang et al., 2013). The exclusively Blattabacterial composition of the microbiome in embryos suggests that this symbiont must be the source of these essential nutrients. However, previous studies characterizing the genome of Blattabacteria inhabiting other species of cockroaches have shown that only the strain belonging to the German cockroach (*Blattella germanica*) possesses the capability to synthesize methionine, one of the amino acids lacking in *D. punctata* milk, in any capacity (Huang et al.., 2012; Kambhampati et al., 2013; López‐Sánchez et al., 2008, 2009; Neef et al., 2011; Patiño‐Navarrete et al., 2013; Sabree et al., 2012, 2009; Tokuda et al., 2013). Consequently, further investigation of this symbiotic relationship is required to understand the role of Blattabacteria during intrauterine development. Sequencing the genome of the *D. punctata* strain of Blattabacteria may reveal the presence of biosynthetic pathways that can provide amino acids required for prenatal development.

In conclusion, we provide a comprehensive survey of the microbial communities of mothers and their developing embryos along with succession of the microbiome community across postnatal development in *D. punctata*. This study provides evidence that, unlike other viviparous insects, there is no transmission of bacteria from mothers to offspring during their 63+ day pregnancy. Surprisingly, we also found no evidence that there is significant bacterial colonization of *D. punctata* during birth or within the few hours immediately following birth. Rather, a majority of the microbiome components are acquired, likely from their environment, throughout the full duration of the first‐instar and melanization period. Further investigation will be required to further elucidate the specific mechanisms underlying nutrient provisioning by Blattabacteria during embryonic development in *D. punctata*, as well as the role of the microbiome during nymphal development.

## CONFLICT OF INTEREST

None declared.

## AUTHOR CONTRIBUTIONS

E.C.J. and J.B.B. conceived the study. E.C.J. designed the experiments and, with guidance from T.L.H., analyzed all data. E.C.J. and M.W.K. collected samples, performed DNA extractions and prepared samples for sequencing. T.L.H. coordinated sample transportation and sequencing. E.C.J. and J.B.B. wrote the paper and T.L.H. edited the paper. E.C.J., M.W.K., T.L.H. and J.B.B. contributed substantially to interpreting the data and developing the manuscript and take full responsibility for the content of the paper.

## Data Availability

Sequence data have been added to the NCBI Sequence Read Archive (SRA) database (PRJNA522760), additional output from analyses using the Nephele implementation of QIIME can be found in Data S1 and Data S2.
