# Supplementary material for: Matrotrophic viviparity constrains microbiome acquisition during gestation in a live‐bearing cockroach, Diploptera punctata
Source: Ecol Evol. 2019 Aug 22;9(18):10601–14. doi: 10.1002/ece3.5580 (PMC6787804; doi:10.1002/ece3.5580)
Supplement: Supplementary file 1 [file ECE3-9-10601-s001.docx]

**Supplemental figure 1.** Scatter plot of the relative abundances of the ten most abundant classes and all other classes combined. Point color indicates class designation while point shape corresponds to the treatment group. The linear model describing the relationship between the two methods is represented by the black trendline (*y =* 0.0092 + 1.1x). *r^2^* = 0.943.

**Supplemental Figure 2.** Percent of total sequencing reads for the 19 most abundant bacterial families in *D. punctata* mothers and embryos. The remaining families are cumulatively represented as “other”. The y-axis represents the percent of total OTUs present in each sample for each family. Each bar represents an individual mother or brood of embryos.

**Supplemental tables can be found in the included excel file.**

**Supplemental table 1.** Rarefaction data using species observed (sobs) generated by mothur

**Supplemental table 2.** Read count of each taxonomic assignment of all mothers and embryos as generated by mothur.

**Supplemental table 3.** OTU count of each taxonomic assignment of all mothers and embryos as generated by mothur.

**Supplemental table 4.** Taxonomic assignment of the 2314 OTU core maternal microbiome at the phylum, class, order, family and genus level.

**Supplemental table 5.** Mother-embryo comparisons using HOMOVA and AMOVA calculations on Yue & Clayton (thetayc) distance calculations generated by mothur.

**Supplemental table 6.** Read count of each taxonomic assignment of all developmental stage samples as generated by mothur.

**Supplemental table 7.** OTU count of each taxonomic assignment of developmental stage samples as generated by mothur.

**Supplemental table 8.** Developmental stage comparisons using linear discriminant analysis (LDA) effect size as generated by mothur.

**Supplemental table 9.** Developmental stage comparisons using HOMOVA and AMOVA calculations on Yue & Clayton (thetayc) distance calculations generated by mothur.

**Supplemental data 1.** Output of the Nephele implementation of QIIME on the mother-embryo data

**Supplemental data 2.** Output of the Nephele implementation of QIIME on the developmental stage data
